# The functions of imitative behaviour in humans

**DOI:** 10.1111/mila.12189

**Published:** 2018-05-22

**Authors:** Harry Farmer, Anna Ciaunica, Antonia F. de C. Hamilton

**Affiliations:** ^1^ Institute of Cognitive Neuroscience University College London London UK; ^2^ Department of Psychology University of Bath Bath UK; ^3^ Institute of Philosophy University of Porto Porto Portugal

**Keywords:** imitation, social cognition, function, mimicry, simulation, social learning

## Abstract

This article focuses on the question of the function of imitation and whether current accounts of imitative function are consistent with our knowledge about imitation's origins. We first review theories of imitative origin concluding that empirical evidence suggests that imitation arises from domain‐general learning mechanisms. Next, we lay out a selective account of function that allows normative functions to be ascribed to learned behaviours. We then describe and review four accounts of the function of imitation before evaluating the relationship between the claim that imitation arises out of domain‐general learning mechanisms and theories of the function of imitation.

## 1 INTRODUCTION

A central topic in the contemporary philosophy of mind, as well as in social neuroscience and developmental psychology, is the study of imitative behaviour. Humans imitate across a wide range of tasks and domains with high fidelity and subtle sensitivity to context, more so than any other species (Subiaul, 2016; Whiten, 2011). Two overarching questions can be asked in our attempts to understand imitation behaviour: Where does it come from? What is it for? In the present article, we focus primarily on the second question, describing different theories of the function of imitation and considering how our current theories of the origins of imitation relate to what we use imitation for.

To preview the structure of this article, we will first consider definitions of imitation and will briefly review the debate on the origins of imitation. We suggest that the weight of evidence supports the claim that imitation arises out of general sensorimotor learning mechanisms (Cook, Bird, Catmur, Press & Heyes, 2014). We then consider what it means to define a function and describe four different accounts of the function of imitation. We briefly review the evidence for each and the kind of studies that could distinguish between different accounts. We conclude with an evaluation of the relationship between the claim that imitation arises out of domain‐general learning mechanisms and the richer theories of the function of imitation.

## 2 DEFINING IMITATION

Imitation behaviour is flexible and variable. People may imitate with their hands, bodies, faces or speech and vocalisations. They may imitate novel actions or familiar actions with high or low fidelity and might act at the same time as another or after a long delay. Some imitation actions might result in the same outcome as the demonstrator, but others might fail or lead to a different outcome. Some imitation behaviours might be consciously controlled, but others seem to occur spontaneously, without awareness. Many different formal definitions of imitation have been given, from Thorndike (1898)‘s focus on imitation for learning to the distinction between emulation, mimicry and true imitation in animal behaviour (Boesch & Tomasello, 1998; Want & Harris, 2002). In the present article, we use the broadest and simplest definition of imitation as follows—we call an action imitation if there is a relationship between the behaviour of a copier and a model, such that observing the movements of the model causes the parts of the copier's body to move in the same way relative to one another as the parts of the model's body (Heyes, 2016a). This is a purely behavioural description that makes no assumptions about intentions or learning or function but, rather, attempts to capture which behaviours can be classified as imitation and which are not. Our aim is to consider the many distinct types of imitation behaviour that humans can engage in and to consider what kind of function (if any) such imitation might have. We will distinguish between types of imitation as needed.

## 3 WHERE DOES IMITATION COME FROM?

A heated debate concerning the origins of imitation behaviour and the underlying neural mechanisms of imitation has taken place over the last 20 or more years. At the core of this debate is the so‐called “correspondence problem”—the problem of matching observed actions in a visual format to performed actions in a motor format in order to perform an imitative action (Brass & Heyes, 2005). In recent years, the debate about the origin of imitation has been closely linked to the question of the origin of the mirror neurons. These neurons were first found in the macaque premotor and parietal cortices (di Pellegrino, Fadiga, Fogassi, Gallese & Rizzolatti, 1992; Gallese, Fadiga, Fogassi & Rizzolatti, 1996), but neuroimaging studies have demonstrated similar activations in humans (Molenberghs, Cunnington & Mattingley, 2012). The key property of mirror neurons is that they fire both when an individual performs a specific action and when that individual observes that action being performed by another. Thus, they are widely accepted to be a suitable neural mechanism for solving the correspondence problem. The questions “Where does imitation come from?” and “Where do mirror neurons come from?” are likely to have closely related answers. In this section, we will briefly outline a number of positions on the origin of mirror neurons, including strong nativist and empiricist accounts as well as hybrid theories of mirror neuron development, which assign a role to both genes and experience.

First, the nativist view of imitation argues that the unique matching properties of mirror neurons have arisen from evolutionary pressure to understand and imitate other people, which has led to genetic mechanisms for linking visual and motor representations of action from before birth (Arbib, 2010; Rizzolatti & Craighero, 2004). This position implies that a difference in the genetic specification of mirror neurons could account for the difference in both the frequency and complexity of imitation seen in humans when compared to other non‐human primates. By positing that mirror neurons are an evolutionary adaptation, the nativist viewpoint is committed to the notion that mirror neurons and, by implication, imitation has a distinct neurocognitive role that enhances reproductive fitness.

In contrast, the strongly empiricist Associative Learning account (ASL account) of imitation focuses on the role of postnatal learning in forging the visual–motor links that support imitation (Cook et al., 2014; Heyes, 2001). The core claim of the ASL account is that mirror neurons develop via the formation of learned associations between observed and executed motor actions that occur during development. This can only occur when the environment provides the infant with a rich repertoire of opportunities to form imitative sensorimotor contingencies (SMCs) (see Cook et al., 2014 for a more detailed review). However, there is no requirement in the ASL account for imitation to have any particular function or role that distinguishes it from other sensorimotor behaviours.

In addition to these two theories at the extreme of the nature–nurture divide, there are several hybrid theories, which take elements of both the genetic and ASL accounts (Del Giudice, Manera & Keysers, 2009; Quadrelli & Turati, 2016). For example, Quadrelli and Turati's (2016) account situates the development of imitation within a neuroconstructivist framework. This account assumes a key role for sensorimotor learning in the development of imitation but emphasises critical periods of early development rather than assuming that infant and adult learning experiences are essentially similar in their effects on mirror neurons. Thus, the neuroconstructivist account is similar to the ASL account in its implications for our understanding of the function of imitation.

The evidence for and against these two theories of the origins of imitation and mirror neurons has been extensively debated over the last decade, and we do not attempt to cover the full debate here. Two key sources of evidence can help us discriminate between the nativist account and the learning account of the origins of imitation. First, do newborn infants imitate? Second, how much can the mirror neuron system change in adulthood? Nativist accounts of mirror neurons have often been supported by appeals to cases of neo‐natal imitation (Meltzoff & Moore, 1989) because if infants only a few days or hours old can imitate, then the mechanism of imitation must be present from before birth. However, a recent longitudinal study with a large sample size and robust measures found no convincing evidence that newborn babies can imitate facial gestures, hand movements or vocalisations (Oostenbroek et al., 2016). This overturns a key argument in favour of the nativist account of the origin of imitation and opens the way for learning after birth to have a strong impact on the creation of mirror neurons (Heyes, 2016b).

In terms of changes in the adult mirror neuron system, several studies suggest that sensorimotor learning can reconfigure the mirror system. For example, in an ingenious experiment, Catmur, Walsh and Heyes (2007) measured mirror system functioning before and after incompatible (“counter‐mirror”) sensorimotor training, during which participants performed index finger movements while observing little finger movements and vice versa. After this training, they found that the motor system activated more strongly when seeing counter‐mirror movements compared to mirror movements, which supports the idea that mirror neuron activation can be altered by exposure to novel sensorimotor correlations. This suggests that if an infant grew up in an environment where mirrors and imitating adults were replaced by dissimilar actions (e.g., foot movements when she moved her hands), then he or she would develop a counter‐mirror system.

Based on these and many other studies, we believe that the overwhelming evidence supports a key role for sensorimotor learning in the origins of imitation, in which our ability to copy actions arises largely from general learning mechanisms. The aim of the present article is to consider the implications of this learning account for our understanding of the function of imitation. In the sections that follow, we explore two important implications. First, if learning is critical for creating SMCs, then the types of SMCs that we have and the types of imitation in which we can engage will depend heavily on the availability of the right information in the learning environment. We explore what this means for different types of action and different types of imitation. Second, if imitation arises from learning (not genes), then it does not make sense to say that evolutionary pressure has selected a brain system “for” imitation. Does this mean that we must be nihilists and say that imitation has no function in human behaviour? Or are there other ways to understand the function of imitation?

## 4 LEARNING DIFFERENT TYPES OF SENSORIMOTOR CONTINGENCIES (SMCs)

The core claim of the ASL account is that people learn imitation in the same way that they learn other SMCs, that is, via a generic associative learning mechanism. There are infinitely many SMCs that an infant could potentially learn, but in practice, he or she learns a subset of this vast space. The constraints imposed by the social and sensorimotor environment play a key role in defining this space. Here, we attempt to explore the space of possible SMCs and consider what might define which ones are learned. Having a clear understanding of the landscape of SMCs will help us consider, in the next section, whether imitative SMCs have a unique place in this landscape and thus could have a unique function.

### 4.1 Imitative SMCs versus other SMCs

**Figure 1:**
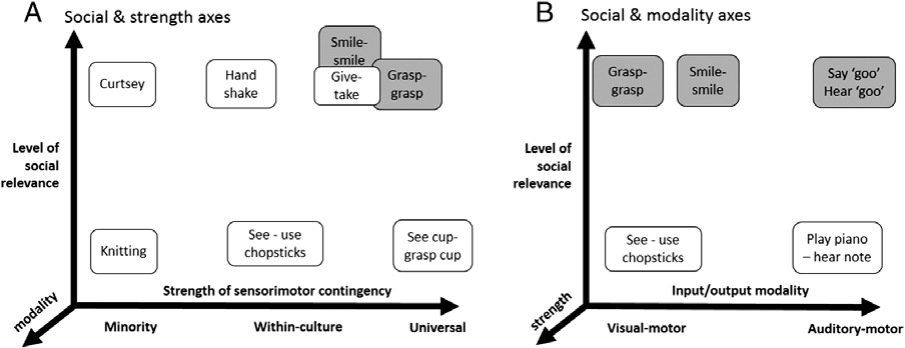
The space of sensorimotor contingencies. People learn a wide variety of sensorimotor contingencies over their lifetime, which we arrange in a 3d space. Panel A shows two axes of this space – social relevance and strength of SMC. Panel B shows a different view of the same space, emphasising social relevance and modality. In both, boxes give examples of SMCs found at different places in the space. Grey boxes indicate SMCs which are imitative

In general, we define an SMC as a link between a sensory stimulus (e.g., a visual image) and a motor action that is learned by sensorimotor associations over the lifetime. For example, seeing the image of a pen and forming the appropriate grasp for writing would form a pen–write SMC. We will begin by describing two dimensions of SMC space (Figure 1a)—first, the social relevance of the stimulus and second, the strength of the learned association. Beginning in the lower right corner of the figure, an example of a strong non‐social SMC might be the affordance of a cup as an object that can be lifted and grasped. It is likely that almost every person in the world has seen, lifted and drank from a cup‐shaped object many times every day over his/her lifetime and has a strong sensorimotor link between the visual image of a cup and the action of grasping it. This can be revealed in priming studies (Tucker & Ellis, 2004). Other non‐social SMCs might be strong in some individuals but weaker in others—for example, the SMC between seeing chopsticks and grasping them to eat would be very strong in people who use chopsticks to eat every day and weak or non‐existent in those who have never used this tool. Thus, within the non‐social realm, SMCs for object use vary in strength according to the cultural and learning environment of different people. Some SMCs for basic actions (e.g., grasping a cup, grasping a rock) are likely to be universal, while others would be found only in specialised communities (e.g., knitting). Thus, we can map the strength of non‐social SMCs on the x‐axis in Figure 1a.

Similarly, within the social domain, there are a variety of possible SMCs. SMCs for imitation of hand actions (e.g., grasp) are likely to be strong because they can be learned largely by self‐observation of one's own hand actions. There is nearly 100% contingency and congruency between observing a visual image of your own hand and performing an action with that hand. Some social SMCs that are non‐imitative may also be strong; for example, the reciprocal action of giving/taking is one that everyone performs many times and is therefore likely to be both strong and social. Experimental data show that seeing a hand extended for a handshake primes the SMC for the contralateral hand that would be the appropriate social response, whereas seeing a hand grasping an apple primes the SMC for the ipsilateral (mirror image) hand (Liepelt, Prinz & Brass, 2010). This implies that the complementary social SMC for shaking hands is as strong as the imitative SMC for grasping. However, we can imagine that other social SMCs might also be culture‐specific, such as a fist bump or a curtsey, and might be found only in people who have learned those actions as part of their culture. This landscape of potential SMCs is illustrated in Figure 1a.

### 4.2 Imitation in different modalities

The introduction to the SMC landscape above focuses on hand action SMCs because this has been the focus of the majority of cognitive studies of imitation. However, SMCs also exist for other types of action, including vocalisations/speech, body actions and facial actions, and it is helpful to contrast these different input and output types (Figure 1b). Visual motor contingencies include hand actions and facial imitation (e.g., smile‐smile) and non‐imitative skills (e.g., chopsticks), while auditory motor contingencies include making/hearing speech sounds and playing instruments (e.g., piano). Different opportunities to learn may apply to each of these.

Self‐observation provides a straightforward mechanism for learning SMCs for hand actions and is present from early in infancy. Not only do newborns move their hands significantly more when they can see them (Van Der Meer, 1997), but more importantly, they actively attempt to control arm movements to maintain their hands in their visual field (Von Hofsten, 2004). Infants at 4 months spent approximately 40% of their waking time viewing their own hand (Ray & Heyes, 2011). Self‐observation also applies to vocal actions—there is a near 100% correlation between performing a vocalisation (e.g., a baby saying “goo”) and the matching auditory input (e.g., hearing her “goo” vocalisation). Thus, for both hand actions and vocal actions, there is strong environmental scaffolding in place for creating imitative SMCs. The challenge faced by the infant in imitation is primarily one of generalisation—how to generalise the view of one's own hands to another person's hands or to generalise one's own sounds to other people's sounds.

In contrast, learning SMCs for facial actions is much more challenging because the self‐observation strategy is less available. When a baby opens her mouth, she does not get direct visual feedback of her mouth opening. A mirror is a possible source, but availability of mirrors is not universal to infants. Instead, it has been suggested that infants learn SMCs for facial actions from seeing other people's imitative actions (Ray & Heyes, 2011), and data on infant–parent behaviour support this possibility. Several developmental studies indicate that infants are born with a predisposition toward face‐related stimuli (Farroni et al., 2005) and gain ample experience in viewing faces (Jayaraman, Fausey & Smith, 2017). More critically, parents have a tendency to mimic their infants, on average, once every minute (Jones, 2009). Messinger, Ruvolo, Ekas and Fogel (2010) found that mothers smile predictably in response to infant smiles from 10 weeks, whereas infant smile initiations become more predictable over the first 6 months. This leads to a process of mutual amplification and the establishment of turn taking around social smiling in early communication (Kärtner, Holodynski & Wörmann, 2013).

It is also worth noting that auditory–visual and auditory–motor contingencies could contribute to helping the infant learn the challenging case of visual–motor contingencies for facial actions. Infants are able to link face shapes to sounds from the age of 4 months, showing a McGurk effect (Burnham & Dodd, 1996). Take the example of an infant saying “goo” followed by the mother's repetition of “goo”. The infant gets information from three modalities—her own motor system as she produces the vocalisation, the sound of her and her mother's vocalisations and the vision of her mother's mouth movements. The co‐occurrence of the three along with the close match between the two sounds will lead to the formation of associative links between her own motor patterns, the vocalisation and the visual representation of her mother's mouth. Thus, multimodal learning, including auditory matching, might help the infant overcome the challenge of learning facial SMCs, although this idea requires further empirical investigation.

The studies of parent–infant interaction described above come from western families, and it is not yet clear whether differences in parenting style within or across cultures could lead to differences in how infants learn imitative SMCs for facial actions. Some claim that parenting styles are culture‐specific and have evolved as adaptations to specific cultural environments (Greenfield, Keller, Fuligni & Maynard, 2003; Keller, 2007). If this is so, then one consequence is that the emergence of imitation and social smiling should be significantly modulated by eco‐cultural constraints. Studies have tested this by comparing infant smiling and imitation across families in Germany, Kenya and Cameroon (Keller, 2007; LeVine et al., 1994). The underlying assumption is that cultural norms and values inform caregivers' social practices and parenting styles, which in turn should lead to different developmental trajectories and outcomes. These studies found that German mothers actively attempt to maintain positive social exchanges during face‐to‐face interactions with their infants. In contrast, Gusii mothers try to keep their babies calm, avoiding positive or negative arousal states by preventing excitement (LeVine et al., 1994). Similarly, in the cultural context of the Nso, a well‐developing child is emotionally neutral, and the ideal way to interact with an infant is to induce and maintain calm contentment (Keller, 2007). However, despite important variations in parenting styles, it is important to note that face‐to‐face interactions that elicit and sustain connectedness were universally employed, making the faces of caregivers a reliable source in establishing SMCs across cultures.

A key implication of this review on facial imitation is that the contingency between performed and observed facial actions exists but may be lower than that for hand or vocal actions. This implies that SMCs should be weaker and more variable for facial actions, which could have implications for function. However, we are not aware of any work that has directly tested the relative strengths of facial and hand action SMCs or of how it would be possible to do this. Further work could also explore the landscape of auditory motor SMCs in more detail, but again, this is beyond the scope of the present article.

### 4.3 Is imitation special?

Given the landscape of SMCs that we sketch out in Figure 1, one key question emerges. Can we say anything special about the imitative SMCs (grey boxes) compared to the other SMCs found within the same landscape (white boxes)? As described above, the core claim of the ASL account is that imitative SMCs are learned in the same way as non‐social SMCs, so acquisition cannot distinguish imitation. Neuroimaging literature shows a strong overlap between the neural mechanisms for social SMCs and non‐social SMCs (Hamilton, 2016). Both engage the core regions of the human mirror neuron system, and there seems little to distinguish these distinct parts of the SMC landscape in terms of brain systems.

These claims of neural and ontological homogeneity across the SMC landscape have important implications for our understanding of possible functions of imitation. Specifically, is it plausible to claim any special function for imitative SMCs? Seeing the SMC landscape allows us to suggest that imitative SMCs may occupy a distinct area of this landscape—they are among the strongest and most social of all possible SMCs. That is, imitative SMCs (at least for hand actions) are very strong because there is ample opportunity to learn them. They are also likely to be a human universal because everyone has the same chance to learn them. In contrast, other social SMCs are likely to be culture‐specific (e.g., curtsey) because only some people in some cultures learn them. If humans were to use a universal, basic form of communication, then universal, imitative SMCs could be a good starting point, while non‐imitative SMCs would not be so appropriate. Thus, the unique position of imitative SMCs in the landscape could give imitation a distinct function unlike other SMCs. We will consider these possible functions in more detail in the next section.

## 5 THE POSSIBLE FUNCTIONS OF IMITATION

### 5.1 Defining function

Before we examine what functions have been ascribed to imitative sensorimotor contingencies, we first need to clarify what we mean by function. For example, Godfrey‐Smith (1993) distinguishes between two distinct notions of functional explanations, both of which he considers to be valid. The first expressed clearly by Cummins (1975) is the causal role effects account of functions (C‐Functions), which hold that the function of a trait is determined by how its effects contribute to the explanation of more complex capacities and dispositions of the system that possesses it. To say that the C‐Function of the heart is to pump blood implies that it is it through the action of pumping of blood that the heart contributes to the operation of the circulatory system. However, one key point to note about C‐Functions is that, on this account, functions lack normative force. This means that there is no way to distinguish between a trait that is present specifically because of the functional role it plays and one that arose due to a lucky accident; if both lead to the same effects in the system, then both possess a C‐Function. This has led many theorists to argue that C‐Functions cannot capture the important sense in which to say that a trait has a specific function is to say that that trait is present because of that function.

This weakness of C‐Functions has motivated many theorists to adopt an alternative selection effects account of functions (S‐Functions) (Godfrey‐Smith, 1993, 1994). This account builds upon an idea first laid out by Wright (1973) and stipulates that for a trait of a system to have a function it must both perform a certain role and be present in the system due to its ability to perform that role. In terms of the heart, for instance, its S‐Function is pumping blood because that is how it contributes to the circulatory system and the heart is present in the system because it has been selected by evolution due to its contribution in pumping blood around the body. While natural selection is the most obvious mechanism that gives rise to S‐Functions, several theorists (e.g., Millikan, 1989; Papineau, 1984) have argued that any process in which a trait aids in the reproduction or replication of its bearer when compared with alternative traits, including cultural and linguistic evolution and “trial and error” learning, can generate S‐Functions. More recently, Garson (2012, 2017) has argued for a more liberal notion of selection in which the S‐Function of a trait can be defined by its contribution to the persistence rather than the replication of its bearer within a population. Garson's account, therefore, allows S‐Functions to be attributed to non‐replicating processes that, nonetheless, involves selection between different alternatives such as neural selection or, in the case of imitation, the selection of specific behaviours.

If we consider the question of the function of imitation within such a framework, we can more clearly distinguish as to what is at stake in this debate and how it relates to the different theories on the origins of imitation outlined above. One point to make is that proponents of the ASL account often speak of divorcing origins from function (Cook et al., 2014; Heyes, 2016a), but it is unclear exactly which of the two meanings of function they have in mind when they say this. Indeed, Cook et al. seem to include both C‐ and S‐Functions in their discussion of the functions of mirror neurons grouping both “what mirror neurons do or what they are for?” as the questions that are relevant for their function. However, on the S‐Function account, such a full divorce of origin and function seems impossible as behaviours come to possess their functions only through their past selection.

Interestingly, Heyes (2014) points towards Tinbergen's (1963) concept of four essential forms of biological explanation, which separates a trait's evolution from its function (sometimes referred to as its survival value). It is important to note that one way that Tinbergen's distinction between evolutionary origin and function can be maintained is that, on Garson's (2012, 2017) liberal notion of S‐Function, it is perfectly possible for imitative behaviour to be assigned an S‐Function based on a selective process other than genetic evolution, for example, via the selection of specific motor commands within the brain. This allows for functions to be assigned to imitative SMCs even on the ASL account of imitation by saying that Tinbergen's evolutionary explanation of imitative SMCs would rely on the importance of strong sensorimotor neural connections for the regulation of one's actions, while the functional explanation would be concerned with the factors that lead to the selective deployment of imitative SMCs during social interaction.

A second key point when considering which version of function is relevant for the current question is the possibility that imitation may have C‐Functions without possessing any S‐Functions. It is possible to imagine that imitation may consistently have an effect when interacting with others despite the fact that imitative behaviour has not been selected by the motor system because of those effects. In such a case, the effect of imitation would simply be the result of a happy accident rather than the reason that imitation occurs. However, this reading brings up the key problem with C‐Functions, which is that, in such a reading, the fact of being a function appears to lack any real explanatory power regarding why imitative behaviour is displayed during social interaction. Therefore, in the rest of this article, we will employ the S‐Function saying that an effect is a function of imitation when it requires not only the fact that imitation causes an effect but also that there is evidence that imitative behaviour is produced to cause that effect. By this, we do not mean that imitation must be consciously used to achieve that effect but merely that there should be evidence of an increased propensity to deploy imitative behaviour in situations in which that effect is desirable. This implies that the key test for any proposed function of imitation is to demonstrate that the occurrence of imitative behaviour is modulated by factors relevant to that function. If we are exploring imitation in terms of neural systems, such modulation might be indicated by interactions between the core mirror neuron system that drives imitation and neurocognitive mechanisms that are, to some extent, specialised for that function (e.g., social affiliation or mentalising about others) rather than mere domain‐general mechanisms, such as attention.

Building on this definition of function, we can now consider what possible functions of imitative SMCs might be compatible with the ASL account of the origins of imitation. There are at least four possible ways to approach this question, and the accounts given below are not all mutually exclusive. The most radical view is to simply deny that imitation has any function and to claim that the SMC landscape is all the same; we term this the “nihilist account”. A second option is that imitation serves mainly the purpose of understanding others' actions via simulation. A third account suggests that imitation has a social–communication role, to send signals of affiliation and allow the reciprocal interactions that sustain and foster social connectedness. Finally, imitation may function primarily for learning and cultural transmission of information. In what follows, we address each viewpoint in turn, highlighting the features and challenges that these theories raise.

### 5.2 The nihilist account

The first prospective candidate is what we will term the nihilistic view of imitation. This view denies that imitative behaviour fulfils any specific function that marks it out from other, more general forms of interpersonal coordination or sensorimotor coordination. That is, the nihilist account claims that imitative SMCs and non‐imitative SMCs are indistinguishable, so whatever functions apply to one must also apply to the other. It is worth noting that the nihilistic interpretation of imitation's function is only plausible on the ASL account of the origin of imitation because other accounts that allow a role for evolution imply that imitation must have a function.

The distinction between C‐Functions and S‐Functions allows us to distinguish between stronger and weaker versions of a nihilist model. In the strong version, imitative SMCs lack even C‐Functions in that they do not lead to any effects on social interaction whatsoever. In the (more plausible) weaker version, while imitative SMCs may lead to some positive social effects, those effects are not the reason why imitation occurs, and therefore, imitation does not possess any S‐Functions. In this weaker account, imitative behaviour might, for example, lead to a strengthening of social bonds between people, but in fact, the generation of such social bonds plays no part in why people imitate others.

A challenge for the weak nihilist account of imitation is to explain the many studies which report that imitation has social consequences (detailed below). This challenge can potentially be met by considering the role of contingency and predictability in social interactions. For example, a contingency explanation would argue that experiencing an effect that is contingent on my action is rewarding because it allows for more efficient associative learning about the relationship between my actions and those of the other person. In such a case, imitation would also be rewarding because this is a sub‐category of contingent action. The key claim here is that any contingent action should be equally rewarding; it does not need to be imitation. Catmur and Heyes (2013) manipulated contingency and similarity independently and found evidence that only contingency matters for prosocial effects. Similarly, Dignath, Lotze‐Hermes, Farmer and Pfister (forthcoming) showed that both the contingency and temporal contiguity of observed and executed actions also modulate affiliation. In contrast, Sparenberg, Topolinski, Springer and Prinz (2012) suggest that effector matching is more important than contingency. One attempt to account for all these effects is to suggest that matching or contingent responses lead to greater fluency effects due to the ease of associative learning (Winkielman & Cacioppo, 2001) and do not demonstrate a specifically social function for imitation.

Similarly, a predictability account of imitation might argue that a key function of the brain is to predict the sensory inputs it receives (Kilner, Friston & Frith, 2007). If I do an action and then another imitates me, his or her action might be easier to predict than if he or she did not imitate me. Thus, the imitative action should lead to lower prediction errors and have positive effects. This possibility is explored further in Hale and Hamilton (2016). Further study to distinguish the exact roles of contingency, similarity and predictability in mediating the consequences of imitation will be very useful in testing the nihilist account and understanding whether the function of imitation differs from other types of SMC.

### 5.3 The simulation account

The simulation account proposes that imitation functions to enhance our own understanding of the behaviour of those we interact with. This explanation for the function of imitation is largely derived from theories that make a link between the simulation theory of mind and the human mirror system (Gallese & Goldman, 1998; Kilner et al., 2007), arguing that mirror system activation is involved in allowing us to understand or predict the actions of others by simulating them using our own motor systems. On this account, the functional role of imitation is situated within the mind of the person performing the imitation, who moves (overtly or sub‐threshold) in order to understand his or her partner but not to send a signal to the partner (Wang & Hamilton, 2012).

One of the most fully developed variants of the simulation account is the interactive–alignment account of dialogue put forward by Pickering and Garrod (2013), which focuses on verbal dialogue as a case of joint action and makes the case that successful dialogue involves the alignment of interlocutors at multiple levels, ranging from situational models (Zwaan & Radvansky, 1998) through lexical choice (Clark & Wilkes‐Gibbs, 1986) to articulatory features such as pitch (Gijssels, Staum Casasanto, Jasmin, Hagoort & Casasanto, 2016), speech rate (Staum Casasanto, Jasmin & Casasanto, 2010) and accent (Adank, Hagoort & Bekkering, 2010). For example, Adank and colleagues have shown that overtly imitating speech in a foreign accent, as opposed to repeating it in one's own accent, enhances comprehension of novel speech heard in that accent. This finding suggests that, at least in the domain of speech, mimicry can play a role in enhancing our understanding of others.

The second area in which robust evidence for the simulation function of mimicry exists is in the imitation of emotional expressions. A large number of studies have demonstrated that participants automatically and subtly mimic the facial expressions of others and that this emotional mimicry has a causal effect on our processing of the emotions of the other person (for recent reviews, see Hess & Fischer, 2014; Wood, Rychlowska, Korb & Niedenthal, 2016). For example, emotion perception was impaired when participants had recently had Botox injections, which prevented the movement of their facial muscles (Neal & Chartrand, 2011). However, negative results have also been reported. Both people with Moebius syndrome (who cannot move their face) and those with restricted face motion can perceive facial emotions (Bogart & Matsumoto, 2010; Kosonogov, Titova & Vorobyeva, 2015). Thus, some argue that facial mimicry is important to the perception of emotion (Wood et al., 2016), while others argue that mimicry plays a small role (Tamietto et al., 2009).

So far, we have shown that the simulation account of imitative function has some support in speech processing and mixed support in emotion processing. Evidence for a role of simulation in understanding hand actions or whole body actions is harder to find. Brain regions linked to simulation are important for understanding actions (Pobric & Hamilton, 2006), but cognitive evidence is weaker. In fact, one of the few studies to look at the causal effect of training imitation on the understanding of others at the level of perspective taking found that training on how to inhibit, as oppose to enhance, imitation led to improved performance in perspective‐taking tasks (Santiesteban et al., 2012). This may be because learning how to inhibit imitation involves increasing the ability to separate others' actions from our own, similar to the need to distinguish another's perspective from our own during perspective taking. Taken together, these findings suggest that, while the simulation account of imitative function may be reasonable in the cases of speech and facial imitation, it is less well supported when applied to the manual and postural movements that are commonly used in studies of imitation.

### 5.4 The social glue account

The account that imitation functions as a “social glue” (Chartrand & Bargh, 1999; Chartrand & Lakin, 2013) focuses particularly on mimicry, that is, imitation of non‐goal‐directed actions, which is not deliberately controlled. It claims that mimicry functions to promote social bonding and affiliation with members of our groups. Two related functions of mimicry can be distinguished within the “social glue” account. First, we could assign mimicry a communicative function, seeing the person who mimics as a “sender” and his or her partner as a “receiver”. A core assumption here is that mimicry is a prosocial signal, which sends a message of affiliation. The second possible functional explanation sees mimicry as a core part of reciprocity, where mutual mimicry between two individuals provides a more basic indicator that the other is a responsive living being (not a machine). Different studies have examined mimicry as a prosocial signal and mimicry as part of reciprocity in different contexts, and we consider each in turn.

To test the possibility that mimicry has a prosocial signalling function, we can look for three types of evidence: first, is mimicry increased when the receiver can see the signal?; second, do receivers respond appropriately when they are mimicked?; third, is the production of mimicry modulated by contexts where we would expect more prosocial signalling to occur?

Addressing the first point, a small but growing number of studies show that mimicry is enhanced when the mimicker is being watched. This has been shown in both tightly controlled reaction time tasks (Wang & Hamilton, 2014; Wang, Newport & Hamilton, 2011) and also more naturalistic contexts (Bavelas, Black, Lemery & Mullett, 1986). Moreover, 5‐year‐olds want to make sure that a model saw their imitation, thus suggesting that their imitative behaviour was produced for the model (Nielsen & Blank, 2011).

Addressing the second point, evidence that people respond positively to being mimicked is also present in several studies, though the overall picture is more mixed. For example, being imitated or mimicked has reliably shown to increase prosocial behaviours in adults, which extend beyond the mimicry interaction (van Baaren, Janssen, Chartrand & Dijksterhuis, 2009). In addition, after being mimicked, participants are more likely to agree with an explicit request for help (Guéguen, Martin & Meineri, 2011) and be more spontaneously helpful. This tendency seems to be in place very early: 18‐month olds are more likely to help pick up pencils after an experimenter has mimicked them (Carpenter, Uebel & Tomasello, 2013). Other infancy studies also show prosocial consequences of imitation. For example, being imitated has been shown to increase gaze engagement between mothers and infants (Field, 1977). Mothers often imitate infants' facial expressions and mirror their level of arousal (Stern, 1985). In turn, infants seem to be able to detect social contingency between their own actions and external responses (Gergely & Watson, 1996). They also seem particularly sensitive to the imitative quality of the response from the interacting partner (Striano, Henning & Vaish, 2006).

In relation to the third point, there is extensive evidence that people produce more imitation when they are in a prosocial context. Indeed, numerous studies show that production of mimicry is modulated by social cues, including motivation to affiliate (Chartrand, Maddux & Lakin, 2006; Lakin & Chartrand, 2003), in‐group membership (Yabar, Johnston, Miles & Peace, 2006) and attractiveness (Karremans & Verwijmeren, 2008; van Leeuwen, van Baaren, Martin, Dijksterhuis & Bekkering, 2009). In contrast, people typically produce less mimicry towards others who they initially dislike (Stel et al., 2010) and towards out‐group members (Bourgeois & Hess, 2008; Johnston, 2002). These results are compatible with the claim that social signals dictate when, who and what to mimic/imitate. According to the social top–down response modulation (STORM) model, people use mimicry as a strategic intervention to change the social world for self‐advancement (Wang & Hamilton, 2012). Importantly, the STORM account of mimicry argues that the core abilities necessary for imitation are instantiated in the mirror neuron system but posits that this system is modulated by social cognitive systems to enable the use of mimicry as a social strategy.

Taken together, these findings suggest that mimicry meets the three criteria set above for having a social‐signalling function—it is produced more when the receiver is watching, and the receiver responds appropriately, and it is produced more in prosocial contexts. However, in real‐life social interactions, people may mimic and imitate one another reciprocally. In contrast to the sender–receiver experimental case, in naturalistic settings, the imitative behaviours unfold in a radically different manner as both partners are agents who dynamically co‐regulate each other's behaviour, and the possibility for influence is always reciprocal. Moreover, imitation is from the outset an interactive and reciprocal phenomenon as infants and caregivers imitate each other. This mutual responding to each other's behaviour functions as a coupling mechanism, which enables both partners to recognise the other as an active and prosocial agent who can interact with them.

Importantly, it is not only the mutuality of these influences that enhances social exchanges but also the open‐endedness and novelty of the dynamic interplay and responsiveness to each other's behaviours. For example, Messinger et al. (2010) demonstrated that infant and mother's behavioural inclinations create from the very beginning a highly dynamic system that seems to aim at reducing predictability. In other words, each partner was most predictable when acting in a context (a state) he or she had created and was less predictable when responding to the other partner's action. This continuous imitative loop may serve a communicative function as reciprocal responsiveness could be regarded as a social signal per se aiming at eliciting and sustaining the social exchanges.

Several studies suggest that young children use imitation to initiate and maintain contact with their pre‐verbal peers. Importantly, being imitated facilitates social engagements in cases where the social skills are impaired, such as in children with autistic spectrum disorders (ASD) (Nadel, 2015). Imitation can act as an interaction strategy for ameliorating the consequences of early social difficulties and as a crucial tool in facilitating social communication and designing potential behavioural interventions in ASD (Escalona, Field, Nadel & Lundy, 2002; Sanefuji & Ohgami, 2011). Given the intricate connection between imitation and sociality, especially in early development, this body of work suggests that imitation may hold a deeply social role, which goes beyond using others' actions for learning via SMCs. In other words, imitation serves not only the previously recognised function of promoting skill acquisition but also, most importantly, the less‐acknowledged communicative function by signalling, eliciting and conveying social information (Nadel & Dumas, 2014).

### 5.5 The cultural learning account

The fourth and final account of imitative function that we will consider is the idea of imitation as a tool for learning and cultural transmission. In this account, the human tendency to imitate plays a vital role in allowing for the vertical transmission of cultural knowledge from one generation to the next (Heyes, 2012;Legare & Nielsen, 2015 ; Shea, 2009). In this account, the human tendency to imitate others plays a key role in the passing down of instrumental skills and social conventions and rituals across the generations of a social group. By doing so, imitation therefore allows for the cumulative cultural development that marks human society out as distinct from that of other animals (Tennie, Call & Tomasello, 2009).

In terms of the selective definition of function we employ in this article, the cultural learning account of imitation could be considered to assign two levels of function to imitative behaviour. The first of these would be the effect of aiding the cultural transmission of specific behaviours that were imitated. On top of this, however, the phenomena of imitation in general could also be said to have a meta‐function of allowing the reliable transmission of behavioural phenotypes. However, as Shea (2009) points out, in order assign the function of cultural transmission to imitation, it is necessary to show that imitation in humans has the traits necessary to act as a mechanism of inheritance. Shea identifies the key traits of any inheritance system as stable high‐fidelity copying, the ability to transmit a wide array of novel variants and the existence mechanisms to prevent “out‐law” copying, that is, copying by non‐kin members, resulting in decreased individual fitness. It is worth noting that the need for the last of these can be reduced by appealing to the adaptive effects of imitation residing at the level of group rather than individual selection (Richerson & Boyd, 2005) or by taking a mimetic account that views the unit of selection as at the level of the produced behaviour rather than the individual. As a discussion about the correct units of cultural evolution is outside the scope of this article, the remainder of this section will focus on the evidence for the first two conditions.

High‐fidelity copying of observed actions is vital to ensure that cultural knowledge is transmitted in a robust enough manner to persist across generations. This is particularly important when the behaviour in question is ritualistic, that is, less related to achieving a goal and more related to the establishment of group norms (Fischer, Callander, Reddish & Bulbulia, 2013). In the case of such rituals, although the purpose of a particular action may be unclear, failure to perform that action correctly could lead to social ostracism from the group and, over the longer term, would prevent the reliable transmission of signs of group membership from one generation to the next. One possible threat to the reliable transmission of cultural norms is individual innovation (Legare & Nielsen, 2015; Shea, 2009), which could lead to too much variation in the actions of model and imitator for the ritual to be replicated faithfully. A second is the use of emulation, copying the goal of an action rather than imitation, the copying of the precise means to perform an action (Tomasello, 2016). The third threat to reliable transmission could be the imitation of movements that were accidental and not desired by the model. Therefore, if imitation has the function of encouraging cultural learning, we should expect to find evidence that people copy the actions of others with minimal deviation from the demonstrator, that they copy the means of performing an action even when other more efficient means of achieving that end exist and that they imitate less if there is reason to believe an action is accidental.

Evidence in favour of the notion that imitation has been selected for high‐fidelity transmission comes from findings suggesting that both children (Lyons, Young & Keil, 2007) and adults (Flynn & Smith, 2012; McGuigan, Makinson & Whiten, 2011) tend to “over‐imitate” the actions of others by imitating steps of an action even when it is clear that those steps are not causally necessary to bring about the goal of the model. This tendency appears to be lacking in other animals, such as chimpanzees (Horner & Whiten, 2005); tends to increase across development (Marsh, Cardinale, Chentsova‐Dutton, Grossman & Krumpos, 2014); and occurs even when the action is judged as causally irrelevant (Kenward, Karlsson & Persson, 2011), suggesting that it is related to the maintenance of social norms rather than a deficiency in the imitator's ability to causally reason about the actions of the model.

Further evidence that imitative behaviour has been selected to promote the transmission of cultural norms comes from findings suggesting that over‐imitation is not purely automatic but that its occurrence can be modulated by context. For example, children over‐imitate more in difficult tasks (Williamson, Meltzoff & Markman, 2008) and are more likely to imitate an adult compared to a peer when learning a novel task but are more likely to imitate a peer when performing a familiar task (Zmyj, Aschersleben, Prinz & Daum, 2012). Other studies have demonstrated that children also discriminate between which model to copy based on factors such as prestige (Chudek, Heller, Birch & Henrich, 2012), familiarity (Corriveau & Harris, 2009) and group membership (Kinzler, Corriveau & Harris, 2011), all of which would play a useful role in ensuring that cultural information was likely to be transmitted reliably within a group. However, despite this evidence, Heyes (2017) has argued that it is currently unclear whether such selective imitation is mediated by domain‐specific mechanisms to promote cultural transmission rather than domain‐general mechanisms such as attention. If it were the latter, then the reasons to suppose that imitation was selected to allow for cultural transmission would be weakened. For this reason, it is important for future research in this field to include asocial attentional controls in order to rule out domain‐general explanations.

The second key trait that Shea (2009) argues any successful cultural transmission system must have is the ability to transmit a wide variety of novel variants. Somewhat counter‐intuitively, this condition suggests that the ASL account of imitation's origins may be better placed to explain how imitation could act as a mechanism of cultural evolution than does the genetic account. This is because the genetic account assumes that the basic operation of the mirror neuron system relies on a foundation of “pre‐wired” connections between specific visual and motor actions, which may later be affected by learning. However, such a pre‐wired system would suggest somewhat less plasticity in terms of the novel gestures that could be passed on than the ASL alternative, which can accommodate any number of SMCs. This raises the possibility that the imitative abilities that form one of the key mechanisms of cultural learning may themselves develop through a combination of domain‐general learning and immersion, via social interaction, in our cultural milieu (Heyes, 2012; Heyes & Frith, 2014; Tennie et al., 2009).

## 6 CONCLUSION: CONTRASTING ORIGINS AND FUNCTIONS

In the sections above, we have reviewed evidence for four different accounts of the functions of human imitation: (a) a nihilist account, (b) a simulation account, (c) a social glue account and (d) a cultural learning account. While the nihilist account rules out the other three options, it is possible that simulation, social glue and learning mechanisms can all co‐exist, with distinct functions coming to the fore in different contexts. However, it is important to consider how these theories of the function of imitation relate to claims about the origins of imitation.

As described above, we agree with ASL theorists in saying that imitation does not depend on an innate brain mechanism, but rather, it arises from SMCs learned via both direct self‐observation and direct world observation. The core aim of the present article is to consider if this ASL account also requires a deflationary view according to which imitation is functionless or if richer understandings of the function of imitation are feasible.

We believe there are three reasons to suggest that they are. First, imitation occupies a unique corner of the SMC landscape, being both highly social and strongly learned in all individuals. This gives imitation the potential to take on specific functions that might not be shared with other SMCs. Second, the environment that provides the means of creating SMCs is from the outset not only physical but also social. Hence, the nihilist view that that imitative SMCs and non‐imitative SMCs share the same functions might miss an important distinction between sensorimotor learning from observing or interacting with (physical) objects and learning from observing and interacting with (minded) subjects. Unlike the former, the latter requires and engenders mutual regulation of interpersonal interaction and imitative dynamics. Thus, both learning imitation and producing imitation involves interacting with other people, and this very socialness may influence the domain‐general learning mechanisms that enable imitation.

This leads to the third reason—that the evidence reviewed above demonstrates that imitation is not a behaviour that occurs in isolation or without control. Rather, imitation is heavily modulated by social factors, including gaze, affiliative goals and many other social factors. Neuroimaging studies similarly show that mechanisms of imitation are closely linked to and modulated by social brain systems (Wang, Ramsey & Hamilton, 2011) and reward systems (Trilla Gros, Panasiti & Chakrabarti, 2015). Such modulation likely reflects the intimate links between mechanisms of imitation and other social mechanisms, which could be built by either learning or genetics and which enable imitation to be used as a tool for simulation, social learning and social connections.

A final observation is that, by adopting a selection account of function, it is possible to assign normative functions to behaviours even when those behaviours arise not as genetically specified adaptations but rather through domain‐general learning processes during individual development. Here, we show the utility of such an account when approaching the possible function of imitation. However, more generally, we hope to have demonstrated that the selective account of function can be of use in the meaningful ascription of function to a wide range of cognitive behaviours.

Our review here also suggests several potential directions for further investigation. It would be useful to distinguish more carefully between the different social functions of imitation in order to determine if and when different types of imitation behaviour serve distinct functions. It would also be valuable to explore the SMC landscape more carefully considering how other well‐learned social actions such as reciprocal actions (e.g., give‐take) relate to imitation and how different learning histories may impact on how different SMCs are used in social contexts. It would be helpful to understand the relationship between imitation and other social mechanisms, including theory of mind, motivation to engage with others and emotion processing. Finally, we suggest that a deeper understanding of the function of imitation would make a valuable contribution to both the social neuroscience and philosophy of interactive and reciprocal behaviour.
